# RETRACTION: lncRNA NONHSAT069381 and NONHSAT140844 Increase in Aging Human Blood, Regulating Cardiomyocyte Apoptosis

**DOI:** 10.1155/omcl/9861976

**Published:** 2026-05-28

**Authors:** Oxidative Medicine and Cellular Longevity

RETRACTION: J. Zhao, X. Lin, H. Sun, et al., lncRNA NONHSAT069381 and NONHSAT140844 Increase in Aging Human Blood, Regulating Cardiomyocyte Apoptosis, *Oxidative Medicine and Cellular Longevity*, 2021, 9465300, 16 pages, 2021 https://doi.org/10.1155/2021/9465300.

The above article, published online on 22nd July 2021 in Wiley Online Library (wileyonlinelibrary.com), has been retracted by agreement between the authors; the journal Editor‐in‐Chief, Jeannette Vasquez‐Vivar; and John Wiley & Sons Ltd. The retraction has been agreed due to figure concerns. Specifically:

⦁ Figure 2b has overlapping regions between KD‐Ctrl and KD‐140844, as noted on PubPeer [1].
⦁ Figure 3b has highly similar image elements with the following articles, as noted on PubPeer [2]:
  ⚬ Figure 5F of (Qi et al. 2019) [retracted], https://doi.org/10.1016/j.omtn.2019.06.010;
  ⚬ Figure 4b of (Zhao et al. 2019) [retracted], https://doi.org/10.1002/jcp.28902;
  ⚬ Figure 2d of (Wang et al. 2019), https://doi.org/10.1186/s12935-019-0808-z;
  ⚬ Figure 7a of (Xu et al. 2020), https://doi.org/10.1002/jcp.29056;
  ⚬ Figure 3b of (Zhang et al. 2019) [retracted], https://doi.org/10.2147/OTT.S214529;
  ⚬ Figure 4e of (Li et al. 2021) [retracted], https://doi.org/10.1111/jcmm.16265,
  ⚬ Figure 3c of (Zhao and Hu 2019) [retracted], https://doi.org/10.1002/jcp.28703.

All of these manuscripts are by different authors. The publisher has confirmed the concerns raised. The results published are therefore considered unreliable, and the article is retracted.

The authors agree with the retraction.
